# Isolation and Purification of Potent Growth Inhibitors from *Piper methysticum* Root

**DOI:** 10.3390/molecules23081907

**Published:** 2018-07-31

**Authors:** Truong Mai Van, Tran Dang Xuan, Truong Ngoc Minh, Nguyen Van Quan

**Affiliations:** Graduate school for International Development and Cooperation, Hiroshima University, Hiroshima 739-8529, Japan; truongmaivan1991@gmail.com (T.M.V.); minhtn689@gmail.com (T.N.M.); nguyenquan26@gmail.com (N.V.Q.)

**Keywords:** *Piper methysticum* L., *Raphanus sativus*, growth inhibitor, flavanone, kavalactone, natural herbicide

## Abstract

*Piper methysticum* (kava) root is known to possess promising weed suppressing activity. The present study was conducted to search for potent plant growth inhibitors from the root of this medicinal pepper plant. The ethyl acetate (EtOAc) extract exhibited the strongest reduction on growth of *Raphanus sativus* (radish) (IC_50_ shoot and root growth = 172.00 and 51.31 µg/mL respectively) among solvent extracts. From this active extract, nine potent growth inhibitors involved in the inhibitory activities of *P. methysticum* root were isolated, purified and characterized by column chromatography (CC), gas chromatography-mass spectrometry (GC-MS), electrospray ionization-mass spectrometry (ESI-MS) and nuclear magnetic resonance (NMR). The six fractions purified by CC included two flavanones: 5-hydroxy-4′,7-dimethoxyflavanone (**C1**) and 5,7-dihydroxy-4′-methoxy-6,8-dimethylflavanone (matteucinol, **C2**) and six kavalactones: 5,6-dehydro-kavain **(C3**), a mixture of kavain and yagonin (**C4**), yagonin (**C5**) and dihydro-5,6-dehydrokavain, 7,8-dihydrokavain, dihydromethysticin and methysticin (**C6**). The amounts of 5-hydroxy-4′,7-dimethoxyflavanone, matteucinol, 5,6-dehydrokavain and yangonin were 0.76, 2.50, 2.75 and 2.09 mg/g dry weight (DW), respectively. The two flavanones **C1** and **C2** exhibited the strongest inhibition on shoot elongation (IC_50_ = 120.22 and 248.03 µg/mL, respectively), whilst the two kavalactone mixtures **C4** and **C6** showed the highest suppression on root growth of *R. sativus* (IC_50_ = 7.70 and 15.67 µg/mL, respectively). This study was the first to report the purification and inhibitory activities of the two flavanones 5-hydroxy-4′,7-dimethoxyflavanone and matteucinol in *P. methysticum* root. The isolated constituents from *P. methysticum* root including the flavanones **C1** and **C2** and the mixtures **C4** and **C6** may possess distinct modes of action on plant growth. Findings of this study highlighted that the combinations of hexane-ethyl acetate by 9:1 and 8:2 ratios successfully purified flavanones and kavalactones in *P. methysticum* root.

## 1. Introduction

Allelopathy is described as a phenomenon by which a plant possesses a natural ability to either inhibit or stimulate the growth of other plants in its vicinity by its biological toxins [1]. Taking advantages of this antagonistic plant–plant interaction, growth inhibitors from many plants have been examined for weed management. Since synthetic herbicides have negative impacts on environment and on human health, natural-based herbicide application is considered as an alternative tool to manage weeds in crop cultivation and establish a sustainable agriculture [2].

*Piper methysticum* G. Frost, belonging to the family *Piperaceae*, is a medical herb native to the South Pacific region [3]. The plant has long been reputed as an indispensable beverage for ceremonial and ritual purposes in Polynesia [3,4]. The consumption of *Piper methysticum* provides positive health effects, such as reduction of anxiety disorders, urinary tract infections and muscle relaxation [5,6]. Besides, root of *P. methysticum* which is commonly left after extraction showed promising inhibition on spontaneous growth of paddy weeds [7]. It was reported that extracts of *P. methysticum* root retard plant growth and possess potent allelochemicals [8,9,10]. Six kavalactones derived from *P. methysticum* root were purchased and examined for herbicidal and antifungal activities [9]. In addition, eight phenolic acids involved in inhibition against the harmful paddy weed *Echinochloa crus-galli* (barnyardgrass), were putatively identified and quantified by high performance-liquid chromatography (HPLC). Principal plant growth inhibitors of *P. methysticum* root belonged to the kavalactone group, which is differ chemically in their pyrone moieties [9]. In addition, whether the roots of *P. methysticum* contain effective growth inhibitors other than kavalactones and phenolic acids, requires further investigation.

This study carried out bioassay-guided trials of *P. methysticum* inhibition on growth of *R. sativus* according to different extracting solvents and fractions separated by column chromatography (CC). Gas chromatography-mass spectrometry (GC-MS), electrospray ionization-mass spectrometry (ESI-MS) and nuclear magnetic resonance (^1^H- and ^13^C-NMR) were used to determine the chemical structures of isolated and bioactive constituents. The inhibitory levels of purified and identified compounds from *P. methysticum* root were also determined and characterized.

## 2. Results

### 2.1. Inhibitory Effect of Different Extracts of P. methysticum Root

Table 1 shows the inhibitory levels of extracts with solvents with different polarity (hexane, chloroform, ethyl acetate, acetone and water). It was found that all extracts exhibited some reduction of the elongations of shoot and root of *R. sativus*. In general, the strength of the inhibitory activities was proportional to the applied doses.

To compare the inhibitory levels among different solvent extracts, IC_50_ values were used. This represents the quantity of the extract needed to suppress 50% of the shoot and root growth of *R. sativus*. Therefore, a lower IC_50_ value indicates a stronger inhibitory activity. The results of inhibition on root and shoot length of *R. sativus* expressed as IC_50_ values are presented in Table 2. Among solvent extracts, the ethyl acetate (EtOAc) extract showed the highest inhibitory levels on root and shoot (IC_50_ = 51.31 and 172.00 µg/mL, respectively). Consequently, the suppressive strength could be ranked in the following order: EtOAc > chloroform > acetone > hexane > water. The greater inhibition of the EtOAc extract implied that it might contain more potent growth inhibitors than the other extracts, therefore, this fraction was selected for further analysis.

### 2.2. Isolation and Purification of Plant Growth Inhibitors by Column Chromatography

Various combinations of solvents for column chromatography (CC) were examined, of which the mixture of hexane (H) and ethyl acetate (E) was the most effective to separate the constituents of the EtOAc extract (data not shown). Finally, the combinations of H:E at ratios 9:1 and 8:2 successfully separated six different fractions identified as **C1**–**C6** (Table 3).

**Figure 1:**
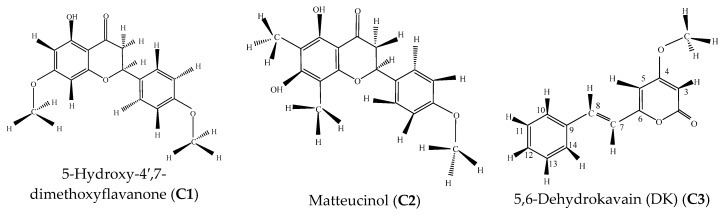

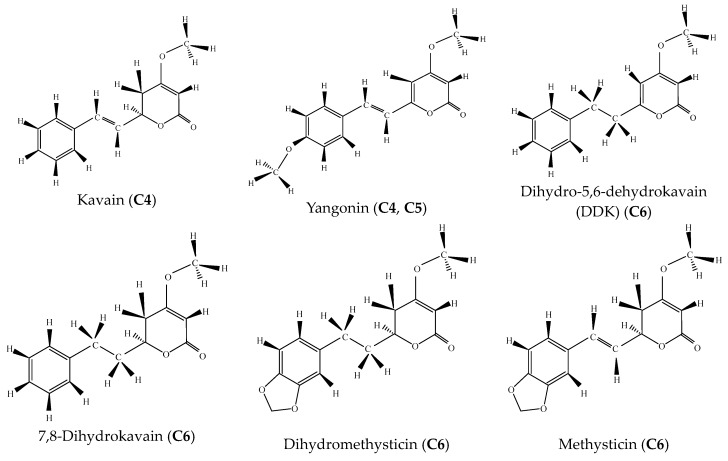
Chemical structures of bioactive constituents from *P. methysticum* root.

Next, the fractions were characterized by GC-MS, ESI-MS and ^1^H- and ^13^C-NMR to identify their chemical components. From 165.9 g powder *Piper methysticum* root, a total of 6.5 g of EtOAc extract was isolated and fractionated by CC (Table 4) into the following fractions: 5,6-dehydrokavain (174.4 mg, **C3**), yangonin (70.3 mg, **C5**), 5-hydroxy-4′,7-dimethoxyflavanone (7.7 mg, **C1**) and matteucinol (54.7 mg, **C2**) (Table 3 and Table 4). The other two solids were mixtures of kavain (59.42%) and yagonin (40.58%) (**C4**) and dihydro-5,6-dehydrokavain (12.78%), 7,8-dihydrokavain (20.44%), dihydromethysticin (54.77%) and methysticin (8.90%) (**C6**, Table 4). The chemical structures of these compounds were elucidated based on their spectroscopic data (Figure 1, Table 4) (Supplementary data, Figures S1–S24). The mixture **C4** afforded the highest yield (518.0 mg), whereas the fraction **C1** provided the minimum amount (7.7 mg) among all fractions.

### 2.3. Quantitative Analysis of Bioactive Compounds from P. methysticum Root

Because the four compounds 5-hydroxy-4′,7-dimethoxyflavanone, matteucinol, 5,6-dehydrokavain and yangonin were purified, they were used as the standards to quantify their contents in *P. methysticum root* by GC-MS (Table 5). Of them, 5,6-dehydrokavain (DK) was the predominant component (2.75 mg/g DW), followed by matteucinol (2.50 mg/g DW) and yangonin (2.09 mg/g DW). The content of 5-hydroxy-4′,7-dimethoxyflavanone was minimum (0.76 mg/g DW).

### 2.4. Inhibitory Activity of the Isolated Compounds on the Growth of R. sativus 

Table 6 showed that all isolated constituents from *P. methysticum* reduced elongation of *R. sativus*, although the levels of inhibition varied among compounds, their mixtures, shoot and root of radish. Among the four purified compounds, both of the two flavanones (**C1**, **C2**) (IC_50_ = 120.22, 248.03 µg/mL, respectively) exhibited much higher inhibitory activities on shoot elongation of *R. sativus* than the two lactones (**C3**, **C5**, IC_50_ = 265.88, 313.82 µg/mL, respectively). The mixtures **C4** and **C6** showed excellent suppression on root growth (IC_50_ = 7.70, 15.67 µg/mL, respectively), as compared to **C3** and **C5** (Table 6). The inhibitory magnitudes of **C4** and **C6** on root growth of *R. sativus* were much stronger than shoot elongation of other kavalactones as well as their mixtures and the two flavanones. The findings in Table 6 suggest that the mixtures of **C4** and **C6** may possess a novel mode of inhibitory action on plant root elongation. However, further trials on the effects of individual compounds in **C4** and **C6** on root growth of different indicator plants including weeds should be performed.

## 3. Discussion

In our previous research, eight phenolic compounds including gallic acid, protocatechuic acid, *p*-hydroxybenzoic acid, *p*-coumaric acid, ferulic acid, salicylic acid, *trans*-*o*-coumaric acid and *trans*-cinamic acid potentially involved in the weed suppressing ability of *P. methysticum* root were identified by high performance-liquid chromatography (HPLC) and thin layer chromatography (TLC) analyses [8]. The most phytotoxic spot visible from TLC reduced the shoot and root elongation of *Echinochloa crus-galli* (barnyard grass) by 80% and 95%, respectively. However, the isolation and purification of plant growth inhibitors other than phenolic acids were not achieved [8]. In another trial, six kavalactones, including methysticin, 7,8-dihydrokavain, 5,6-dehydrokavain, kavain, yangonin, dihydromethysticin were purchased and examined for their inhibitory activities against *Lactuca sativa* (lettuce), barnyard grass and some harmful fungi [9]. Dihydromethysticin was the most potential as it inhibited growth of lettuce by 80.4% by 10 µg/mL, whilst methysticin was the best at reducing barnyard grass emergence (by 66.6%) [9]. Although the efficacy of different extraction solvents on the chemical components in *P. methysticum* root was examined [11], the presence of the different constituents was analysed by only GC-MS [11]. Several attempts on isolation and purification of bioactive constituents have been carried out, but principally related to the medicinal and pharmaceutical properties of kavalactones, using HPLC, GC-MS and common analytical instruments [12,13,14,15,16,17]. Protocols for successfully isolating and purifying bioactive kavalactones as well as bioactive chemicals in *P. methysticum* root have not yet been reported.

The present study investigated the inhibitory effects of different solvent extracts of *P. methysticum* root on the growth of *R. sativus*. It was observed that levels of inhibition on root length were higher than on shoot elongation (Table 2). The phytotoxic effect on plant growth is dependent on the composition and concentration of growth inhibitors [18]. Secondary metabolites from plants including terpenoids, steroids, phenols, coumarins, alkaloids and flavonoids have been documented as allelochemicals [19]. Plant growth inhibitors or allelochemicals inhibit growth of neighboring plants by disturbing their physiological processes such as photosynthesis, respiration, water and hormonal balance [20]. The application of extraction solvents with different polarities is crucial to isolate and purify bioactive compounds in plants [21]. However, many natural products have complicated chemical structures, making their purification processes therefore sophisticated, laborious and time-consuming [22]. The pharmacological properties of *P. methysticum* beverages have attracted many researchers over the past 130 years in order to exploit the biologically active constituents [3,23]. To date, more than 40 compounds have been identified from *P. methysticum* root [24], predominantly belonging to kavalactones, chalcones and flavones and conjugated diene ketones [24], of which, kavalactones accounted for approximately 96% of the lipid extract and 3–20% by dry weight [24,25,26]. However, it was reported that chemotype and kavalactone contents in *P. methysticum* root were controlled by the plant organ, genetics and environmental factors [27]. 

This study found that among solvent extracts, the EtOAc one exhibited the maximal inhibitory effect on the growth of *R. sativus*, as compared to those of chloroform, acetone and hexane (Table 3). Li et al. [28] documented that suitable solvents achieved high yields of potential allelochemicals. Only seven kavalactones, including 5,6-dehydrokavain, kavain, yangonin, dihydro-5,6-dehydrokavain, 7,8-dihydrokavain, dihydromethysticin and methysticin were identified and quantified (Table 4 and Table 5), although a total of 18 kavalactones are known to be found in *P. methysticum* root [11]. The other known kavalactones were absent, thus suggested that they might correlate to much weaker inhibitory activity and be found in other extracts such as hexane, chloroform and water (Table 1 and Table 2). Among the identified constituents, though the flavanones **C1** and **C2** and the individual kavalactones **C3** and **C5** showed stronger inhibition, the mixtures of kavalactones in **C4** and **C6** were the most potent, as they inhibited root elongation of *R. sativus* by IC_50_ = 7.70 and 15.67 µg/mL, respectively (Table 6). The modes of action of the mixtures **C4** and **C6** on different indicator plants including weeds should be further investigated to examine whether they are actually potent enough for the development of novel herbicides. 

In this research, the flavanone **C1** was previously identified by GC-MS [9] and the flavanone **C2** matteucinol was detected for the first time in *P. methysticum* root. This research successfully purified and quantified the two compounds and reported their stronger inhibitory activities than those of other kavalactones on growth of *R. sativus* (Table 6). Compound **C1** (5-hydroxy-4′,7-dimethoxyflavanone) was reported as an antifungal and antioxidant substance in Argentinean propolis and some other herbal plants such as *Piper caninum* and *Combretum zeyheri* [29,30,31], while no information about biological activities of matteucinol was documented. To date, the mechanisms of flavanones other than allelopathy remain vague. It might be that their interference induces cell growth inhibition, ATP production disturbances and hinders the proper functioning of auxins [32].

The findings in Table 3 indicated that the combination of hexane-ethyl acetate at 9:1 and 8:2 ratios successfully yielded the pure flavanones **C1** and **C2** and kavalactones (**C3**, **C5** and mixtures of **C4** and **C6**). The mixture of kavain and yangonin in **C4** and four kavalactones including dihydro-5,6-dehydrokavain, 7,8-dihydrokavain, dihydromethysticin and methysticin in **C6** needed further purification by CC with a more effective combination of solvents. As *P. methysticum* is a Pacific herbal remedy which is highly recommended as a treatment for diseases related to nervous disorders such as anxiety, insomnia and stress [33], although the consumption of kava-containing products has been suspected of causing liver damage in a few cases [34,35,36,37]. In contrast, *P. methysticum* was also reported to be non-toxic to the liver, and it may even possess liver protective properties [38,39]. The protocols for purification of bioactive chemicals in *P. methysticum* root including flavanones and kavalactones developed by this study should prove useful to exploit more effectively the biological activities of this tropical herb. *Alpinia zerumbet* which also possesses kavalactones including DK and DDK [12,40,41,42,43] may be utilized these protocols to isolate potent flavanones and other kavalactones from this medicinal plant. The consumption of *A. zerumbet* as a dietary supplement which includes the bioactive constituents DK and DDK by Okinawans was recorded in a study of the longevity of Japanese people living in islands of the Ryukyus [44]. As DK and DDK are the two kavalactones [9,11,40,41,42,43,44], whether the use of *P. methysticum* root as a traditional beverage contributes to the health and longevity of Polynesian and Pacific islanders needs further investigation.

## 4. Materials and Methods

### 4.1. Plant Materials

#### 4.1.1. *Piper methysticum* Root

Commercial dried *P. methysticum* root was purchased from Kava King Products, Inc. (Ormond Beach, FL, USA) on 2 May 2017. The root powder was stored in the freezer at the temperature of −20 °C prior to extraction.

#### 4.1.2. Tested Plants

Seeds of *R. sativus* were purchased from Taki Co. Ltd. (Kyoto, Japan). Empty and unhealthy seeds were removed by floating in tap water. After air-drying, the selected seeds were treated with 0.1% sodium hypochlorite for 30 min and then washed by sterile water in triplicate.

### 4.2. Preparation of Plant Extract

Dried *Piper methysticum* root powder (907.2 g) was extracted with 3 L of acetone solvent for 2 week at room temperature. After filtration using filter paper (Whatman No.5C, 110 mm), the residue was evaporated to dryness by a rotary evaporator (SB-350, EYELA, Tokyo, Japan) at 45 °C and yielded 49.39 g of crude acetone extract. An amount of 1.5 g the acetone crude extract was dissolved with methanol (MeOH) to enhance the solubility in bioassays and different dilutions (50, 250 and 500 µg/mL) were thus obtained. The remaining acetone crude extract (47.89 g) was dissolved in 100 mL distilled water and fractionated subsequently with hexane, chloroform and ethyl acetate. This resulted in 0.42 g of hexane (0.9%), 46.86 g of chloroform (99.94%), 2.6 g of EtOAc (5.54%) and 0.34 g of water (0.73%) extracts. All extracts were temporarily stored at −5 °C in the dark before the bioassays.

### 4.3. Inhibitory Activity of P. methysticum Root Crude Extracts

Inhibitory assays were performed to measure the effects of various concentrations of *P. methysticum* root extract on *R. sativus* growth inhibition. Three dilutions (50, 250 and 500 µg/mL) of each crude extract were prepared for this study. After that, each ten surface-sterilized seeds were placed in a Petri dish lined with a filter paper moistened with 200 mL of each tested solution. Water was used as a control. Treatments were designed randomly with three replicates. The *R. sativus* seed growth was observed under a condition of 25/23 °C temperature over photoperiod 12/12 h day/night inside a growth chamber (Biotron NC system, Nippon Medical & Chemical Instrument, Co. Ltd., Osaka, Japan). After 5 days, root length and shoot height were recorded to determine the inhibitory levels on of seedling growth and expressed by IC_50_ values. The extract showed the strongest inhibition on the germination and growth of *R. sativus* was further fractionated.

### 4.4. Isolation and Purification of Bioactive Compounds

#### 4.4.1. General Experimental Procedures

Column chromatography (20 mm diameter × 500 mm height, Climbing G2, Tokyo, Japan) was performed using silica gel (size Ǻ 60, 200–400 mesh particle size, Sigma-Aldrich, Tokyo, Japan). Thin layer chromatography (TLC) was conducted by TLC plates with layer thickness was 0.25 mm. The TLC spots were observed by spraying with EtOAc:H_2_SO_4_ (95:5) followed by heating at 100 °C in an oven (Laboratory Convection Oven MOV-212F D, SANYO, Gunma, Japan) for 3 min. GC-MS analysis was run on a JMS-T100 GCV system (JEOL Ltd., Tokyo, Japan). The compound identification was carried out by comparing the mass spectral fragmentation patterns of the samples with the mass spectral libraries of JEOL’s GC-MS Mass Center System Version 2.65a software. The electrospray ionization-mass spectrometry (ESI-MS) experiments were performed in a LTQ Orbitrap XL mass spectrometer (Thermo Scientific, Bremen, Germany). The nuclear magnetic resonance spectra (^1^H-NMR at 400.13 Hz and ^13^C-NMR at 100.612 Hz) were recorded with a model 500 spectrometer (Bruker Yokohama, Japan) with samples dissolved in CDCl_3_. 

#### 4.4.2. Isolation, Purification and Identification by CC, GC-MS, EIS-MS, ^1^H- and ^13^C-NMR

Dried powder of *Piper methysticum* root (165.9 g) was extracted with a solvent system of acetone-acetonitrile (4:1) for one week at room temperature. The extract was then filtered and dried under reduced pressure using a rotary evaporator at 45 °C. The obtained crude extract (9.4 g) was suspended in distilled water and partitioned in a separatory funnel with hexane (H) and EtOAc (E). The most active EtOAc extract was evaporated to yield 6.5 g of crude extract. After thin layer chromatography (TLC) analysis, the EtOAc extract was chromatographed on a column (20 mm diameter × 500 mm height, Climbing G2, Tokyo, Japan) over silica gel (size Ǻ 60, 200–400 mesh particle size, Sigma-Aldrich, Tokyo, Japan). The elution was conducted using sequential solvent systems of increasing polarity (*n*-hexane-EtOAc 10:0–0:10 *v*/*v*), followed by MeOH 100%. Similar fractions (frs.; each of 100 mL) as observed by TLC were pooled together and concentrated to dryness using a rotary evaporator. Six fractions crystallized at room temperature, of which, four were pure compounds and other were mixtures. All isolated compounds and the mixtures were analysed and confirmed by GC-MS and ESI-MS, ^1^H-NMR and ^13^C-NMR to determine their chemical structures. 

*5-Hydroxy-4′,7-dimethoxyflavanone* (**C1**). Compound **C1** was isolated as a white powder from the combined fractions 14–17, which were eluted with 9:1 H-E. This compound was purified after eluting by hexane and filtering through a filter paper to remove contaminants. The purity of the substance was determined to be 93.28% by GC-MS analysis (Supplementary Material, Figure S1). Its molecular formula was identified to be C_17_H_16_O_5_ based on ESI-MS: *m*/*z* 301.1 [M − H]^+^ (Supplementary Material, Figure S2). The identification as 5-hydroxy-4′,7-dimethoxyflavanone (Figure 1) was obtained by analysis of its ^1^H- and ^13^C-NMR spectra and literature data [34]. ^1^H-NMR δ (ppm): 12.8 (1H, s); 7.78 (2H, dd, 8 Hz, 4 Hz, H2′ and H6′); 6.97 (2H, dd, 8 Hz, 4 Hz, H3′and H5′); 6.52 (1H, s, H3); 6.43 (1H, d, 4 Hz, H8); 6.32 (1H, d, 4 Hz, H6); 3.86(3H, s); 3.85 (3H, s).^13^C-NMR δ (ppm): 163.9 (C2), 104.2 (C3), 182.4 (C4), 162.0 (C5), 98.0 (C6), 165.3 (C7), 92.5 (C8), 157.6 (C9), 105.4 (C10), 123.4 (C1′), 127.9 (C2′), 114.4 (C3′), 162.5 (C4′), 114.4(C5′), 127.9 (C6′), 55.8 (C6′–OCH3), 55.5(C7–OCH3). (Supplementary Material, Figures S3 and S4).

*5,7-Dihydroxy-4′-methoxy-6,8-dimethylflavanone* (**C2**). Compound **C2** was precipitated as a yellow powder from fractions 20–26 which were eluted with 9:1 H-E. Hexane was used to purify this compound. The molecular formula of compound **C2** was identified as C_18_H_18_O_5_ based on ESI-MS: *m*/*z* 314.12 [M − H]^+^ while the purity was 90% using GC-MS analysis (Supplementary Material, Figure S6). The identification as 5,7-dihydroxy-4′-methoxy-6,8-dimethylflavanone or matteucinol (Figure 1) was obtained by analysis of its ^1^H- and ^13^C-NMR spectra and literature data [45]. ^1^H-NMR δ (ppm): 2.03(3H, s, 6-CH3), 2.05 (3H, s, 8-CH3), 2.78 (1H, dd, *J* = 17 Hz, 3 Hz, H-3β), 3.03 (1H, dd, *J* = 17 Hz, 13 Hz, H-3α), 3.83 (3H, s, OCH3), 5.32 (1H, dd, *J* = 13 Hz, 3 Hz, H-2), 6.95 (2H, d, *J* = 9 Hz, H-3′,5′), 7.39 (2H, d, *J* = 9 Hz, H-2′,6′), 12.29 (1H, s, chelated 5-OH). ^13^C-NMR δ (ppm): 6.9 (6-CH3), 7.6 (8-CH3), 43.1 (3α, 3β), 55.2 (OCH3), 78.2 (C-1), 102.3 (C-8), 102.7 (C-10), 103.5 (C-6), 114.0 (C-3′,5′), 127.4 (C-2′,6′), 131.0 (C-1′), 157.7 (C-9), 158.8 (C-5), 159.6 (C-4′), 162.1 (C-7), 196.5 (C-4) (Supplementary Material, Figures S6–S12). 

*5,6-Dehydrokavain* (**C3**). Compound **C3** was isolated as pale-yellow needles from fractions 11–15 which were eluted with 8:2 H-E. Hexane was also used to purify this compound with the purity being evaluated as 94.66% by GC-MS (Supplementary Material, Figure S13). According to ESI-MS data (*m*/*z*: 229.1 [M − H]^+^, Supplementary Material, Figure S14), the molecular formula of compound **C3** was determined as C_14_H_14_O_3_. The ^1^H- and ^13^C-NMR results were interpreted to match 5,6-dehydrokavain (Figure 1) by compared with spectroscopic data in the literature [12]. ESI-MS: 229.1 [M + H]^+^; ^1^H-NMR δ (ppm): 3.87 (3H, s, MeO-), 5.62 (1H, d; *J* = 2.2 Hz, 3-H), 6.24 (^1^H, d; *J* = 2.2 Hz, 5-H), 6.86 (1H, d; *J* = 16 Hz, 7-H), 7.43 (1H, d; *J* = 16 Hz, 8-H), 7.36–7.59(5H, m, aromatic); ^13^C-NMR (δ (ppm): 56.61 (MeO-), 89.50 (C-3), 102.73 (C-5), 120,12 (C-7), 128.10 (C-14), 128,44 (C-10), 129,41 (C-13), 129.52 (C-11), 130.23 (C-12), 136.31 (C-9), 136.74 (C-8), 160.55 (C-2), 166.63 (C-6), 173.34 (C-4) (Supplementary Material, Figures S15 and S16).

*Yangonin* (**C5**). Fractions 48–55 were eluted with a solvent system of 8:2 H-E to yield 70.3 mg of yangonin (as light yellow needles). Its molecular formula was established to be C_15_H_14_O_4_ on the basis of ESI-MS: *m*/*z* 259.1 [M − H]^+^ (Supplementary Material, Figure S20). The combined fractions were eluted with a 6:4 H-E solvent mixture to obtain the pure compound. The purity of yangonin reached 93.64% according to GC-MS result (Supplementary Material, Figure S19). The compound was identified as yangonin (Figure 1) by comparison between its ^1^H- and ^13^C-NMR spectra with the spectroscopic data reported in the literature [46]. ^1^H-NMR δ (ppm): 5.45 (d, *J* = 2.1 Hz, H-3); 5.87 (d, *J* = 2.1 Hz, H-5); 6.43 (d, *J* = 16.0 Hz, H-7); 7.43 (d, *J* = 16.0 Hz, H-8); 7.42 (d, *J* = 8.8 Hz, H-10 and H-14); 6.88 (d, *J* = 8.8 Hz, H-11 and H-13); 3.82 (s, 4-OMe); 3.79 (s, 12-OMe). ^13^C-NMR δ (ppm): 164.1 (C-2), 88.3 (C-3), 171.2 (C-4), 100.4 (C-5), 159.0 (C-6), 116.3 (C-7), 135.4 (C-8), 127.9 (C-9), 1289 (C-10), 114.3 (C-11), 160.7 (C-12), 114.3 (C-13), 128.9 (C-14), 55.8 (4-OMe), 55.3 (12-OMe) (Supplementary Material, Figures S21 and S22).

#### 4.4.3. Chemical Identification and Quantification

##### GC-MS Analysis

To elucidate the name, structure and chemical properties of the identified constituents, GC-MS analysis was performed. One µL aliquot of each compound was diluted in chloroform to obtain 1000 µg/mL concentration. After that, the solvents were injected into the GC-MS system equipped with a DB-5MS capillary column, 30 m in length, 0.25 mm internal diameter and 0.25 µm in thickness (Agilent Technologies, J & W Scientific Products, Folsom, CA, USA). The data were recorded using helium as carrier gas. At the beginning of the program, the GC oven temperature program was set up at 50 °C without any hold time, then increased to 300 °C at a rate of 10 °C/min and finally held for 30 min at 300 °C. The injector and detector temperature were set at 300 °C and 320 °C, respectively, with a mass scan range from 29 to 800 amu. The compound identification was carried out by comparing the mass spectral fragmentation patterns of the samples with the mass spectral libraries of JEOL’s GC-MS Mass Center System Version 2.65a and standards.

##### Quantification of Growth Inhibitors of *P. methysticum* Root

Four pure compounds **C1**, **C2**, **C3**, and **C5** were used as standards for the quantitative analysis. Those compounds were dissolved in chloroform to obtain different concentrations (10, 25, 50 ppm). The quantification was performed using similar GC conditions between the standards and samples. The retention time and areas of the standards and samples were compared to obtain standard curves (*r*^2^ > 0.9). The content value of quantified compounds was expressed in milligrams per gram of dry weight (mg/g DW).

##### ESI/MS Analysis

In ESI/MS analysis, the chemicals were solubilized in chloroform-d (CDCl_3_) to obtain a concentration of 50 µg/mL. All measurements were conducted on mass spectrometer with electrospray ion source. Negative/positive ion mode was used with a capillary temperature of 140 °C (120 °C for S2) and a spray voltage of 3.0 kV (2.7 kV for S2). The measurements were performed in the positive mode with an ion spray voltage of 3000 V and a capillary temperature of 350 °C. Data were collected in full scan mode within the range 280 to 1000 *m*/*z*.

##### NMR Analysis

The samples were dissolved in chloroform-d (CDCl_3_). The spectroscopic data were compared with those of literature to identify the structure of isolated organic compounds.

### 4.5. Inhibitory Activity of Isolated Compounds in Bioassays

Different dilutions (5, 10, 50, 100, 250 and 500 µg/mL) of isolated compounds and their mixture distilled in MeOH were prepared to check the germination rate of *R. sativus* seeds. All test plant seeds were soaked in distilled water to remove the empty and undeveloped seeds. Each five remaining seeds were sown in each 12 well-plate (22.1 mm diameter × 35 mm height) with filter paper moistened by 100 mL test compound solvent in triplicate. Methanol was used as the controls. After that, all test plates were kept for 5 days in a growth chamber set at 25 °C ± 2 °C with 8/16 h day/night cycle. The root lengths and shoot heights were measured and the concentration reducing 50% of seedling growth was calculated (IC_50_). 

### 4.6. Statistical Analysis

The statistical analyses were implemented by Minitab^®^ 16.2.3 (copyright © 2012 Minitab Inc., Philadelphia, PA, USA). Mean and standard deviation values were evaluated using one-way ANOVA. Turkey’s test was applied to compare significant differences between treatment, control and standard data various results at *p* < 0.05.

## 5. Conclusions

The findings of this study revealed that *P. methysticum* roots contain a number of potent plant growth inhibitors. The use of combinations of hexane-ethyl acetate at 9:1 and 8:2 ratios in column chromatography resulted in the successful isolation and purification of two flavanones **C1** and **C2**, of which the **C2** (matteucinol) was detected for the first time in *P. methysticum* root and kavalactones **C3**–**C6**, of which the **C2** (matteucinol) was the first time detected in *P. methysticum* root. The ethyl acetate extract showed the strongest inhibitory level as compared with hexane, chloroform, acetone and water ones. The isolated flavanones **C1** and **C2** and kavalactones (**C3**–**C6**) showed reversed different levels of activity on elongation of shoot and root of *S. sativus*, suggesting that they may possess distinct modes of action. This study established an efficacious extraction and purification protocol for the bioactive constituents, principally kavalactones and flavanones in *P. methysticum* roots, to aid effective exploitation of the potential use of this herbal plant for the development of natural herbicides as well as medicines and pharmaceutics. 

## Figures and Tables

**Table 1:** Effect of different extracts of *Piper methysticum* root on growth of *R. sativus*.

Extract	Concentration (µg/mL)	Shoot Inhibition (%)	Root Inhibition (%)
Hexane	50	22.62 ± 3.15 ^d^	28.00 ± 3.73 ^i^
	250	55.33 ± 3.06 ^c^	70.54 ± 3.82 ^d^
	500	68.67 ± 3.06 ^b^	89.52 ± 3.36 ^bc^
Chloroform	50	25.40 ± 2.75 ^d^	36.67 ± 3.51 ^g^
	250	74.00 ± 2.61 ^ab^	88.48 ± 3.94 ^bc^
	500	81.00 ± 3.61 ^a^	98.57 ± 3.43 ^a^
EtOAc	50	24.79 ± 3.39 ^d^	39.33 ± 3.76 ^fg^
	250	78.33 ± 2.08 ^ab^	94.87 ± 3.75 ^ab^
	500	83.67 ± 4.51 ^a^	99.05 ± 3.83 ^a^
Acetone	50	24.13 ± 4.29 ^d^	36.11 ± 3.47 ^gh^
	250	68.67 ± 3.12 ^b^	85.62 ± 2.72 ^c^
	500	78.00 ± 2.00 ^ab^	95.86 ± 3.21 ^ab^
Water	50	23.29 ± 4.02 ^d^	29.00 ± 3.00 ^hi^
	250	30.67 ± 2.31 ^d^	44.83 ± 3.16 ^ef^
	500	51.33 ± 7.02 ^c^	50.48 ± 3.18 ^e^

Values represent means ± SD (standard deviation). Values in a column with different letters are significantly different (*p* < 0.05).

**Table 2:** Effect of *P. methysticum* root extracts on growth of *R. sativus* (IC_50_ values).

Extracts	Inhibitory Levels, IC_50_ (µg/mL)	
	Shoot	Root
Hexane	278.60 ± 19.30 ^b^	173.70 ± 15.44 ^b^
Chloroform	181.09 ± 8.64 ^c^	81.40 ± 26.80 ^c^
Ethyl acetate	172.00 ± 25.20 ^c^	51.31 ± 0.23 ^c^
Acetone	206.36 ± 1.88 ^bc^	90.50 ± 21.00 ^c^
Water	483.80 ± 71.70 ^a^	420.30 ± 53.20 ^a^

Values represent means ± SD (standard deviation); Values in a column with different letters are significantly different (*p* < 0.05).

**Table 3:** Fractions isolated from *Piper methysticum* root by column chromatography.

Fraction	Solvent Combination and Fraction	Weight (mg)
**C1**	Crystal in H-E 9:1 (fractions 14–17)	7.7
**C2**	Crystal in H-E 9:1 (fractions 20–26)	54.7
**C3**	Crystal in H-E 8:2 (fractions 11–15)	174.4
**C4**	Crystal in H-E 8:2 (fractions 32–47)	518.0
**C5**	Crystal in H-E 8:2 (fractions 48–55)	70.3
**C6**	Crystal in H-E 8:2 (fractions 69–74)	49.0

H: hexane; E: ethyl acetate.

**Table 4:** Identification of bioactive compounds from EtOAc extract of *P. methysticum* root by GC-MS, ESI-MS and ^1^H- and ^13^C-NMR.

Fraction	Retention Time	Peak Area (%)	Compounds	Chemical Formula	Molecular Weight	Chemical Class
**C1**	23.40	93.28	5-Hydroxy-4′,7-dimethoxyflavanone	C_17_H_16_O_5_	300	Flavanone
**C2**	25.37	90.00	Matteucinol	C_18_H_18_O_5_	314	Flavanone
**C3**	20.80	94.66	5,6-Dehydrokavain (DK)	C_14_H_12_O_3_	228	Kavalactone
**C4**	20.07	59.42	Kavain	C_14_H_14_O_3_	230	Kavalactone
	23.37	40.58	Yangonin	C_15_H_14_O_4_	258	Kavalactone
**C5**	23.34	93.64	Yangonin	C_15_H_14_O_4_	258	Kavalactone
**C6**	16.25	12.78	Dihydro-5,6-dehydrokavain (DDK)	C_14_H_14_O_3_	230	Kavalactone
	18.35	20.44	7,8-Dihydrokavain	C_14_H_16_O_3_	232	Kavalactone
	22.45	54.77	Dihydromethysticin	C_15_H_16_O_5_	276	Kavalactone
	23.40	8.90	Methysticin	C_15_H_14_O_5_	274	Kavalactone

**Table 5:** Quantity of purified compounds from *P. methysticum* root.

Fractions	Retention Time	Compounds	mg/g DW
**C1**	23.38 ± 0.01	5-Hydroxy-4′,7-dimethoxyflavanone	0.76
**C2**	25.36 ± 0.02	Matteucinol	2.50
**C3**	20.65 ± 0.01	5,6-Dehydrokavain	2.75
**C5**	23.34 ± 0.01	Yangonin	2.09

DW: dry weight.

**Table 6:** Inhibitory activity of isolated compounds from *P. methysticum* root on growth of *R. sativus* (IC_50_ values).

Fractions	Compounds	IC_50_ (µg/mL)	
		Shoot	Root
**C1**	5-Hydroxy-4′,7-dimethoxyflavanone	120.22 ± 14.64 ^e^	-
**C2**	Matteucinol	248.03 ± 5.43 ^d^	-
**C3**	5,6-Dehydrokavain (DK)	265.88 ± 19.78 ^c^^,^^d^	375.33 ± 11.93 ^a^
**C4**	Kavain; yangonin	457.18 ± 28.64 ^a^	7.70 ± 1.57 ^b^
**C5**	Yangonin	313.82 ± 0.68 ^b,c^	365.00 ± 44.00 ^a^
**C6**	Dihydro-5,6-dehydrokavain (DDK); 7,8-dihydrokavain; dihydromethysticin; methysticin	360.19 ± 20.37 ^b^	15.67 ± 5.13 ^b^

Values represent means ± SD (standard deviation). -: measurements were not conducted. Values in a column with different letters are significantly different (*p* < 0.05).

## Supplementary Materials

The following are available online, Figures S1–S24 are provided.
